# Enhancing CPR training and installation of defibrillators in public places – A solution to tackle sudden cardiac arrest

**DOI:** 10.1016/j.resplu.2023.100396

**Published:** 2023-05-19

**Authors:** Tarun Kumar Suvvari, Aditya Lopinti, Charmi Dedeepya Boddapalli

**Affiliations:** Rangaraya Medical College, Kakinada, Andhra Pradesh, India; Government Medical College, Srikakulam, Andhra Pradesh, India; Konaseema Institute of Medical Sciences, Amalapuram, India

Dear Editor,

We read the article “More than 302 million people reached and over 2,200,000 trained in cardiopulmonary resuscitation worldwide: The 2021 ILCOR World Restart a Heart initiative” Published in your journal. The authors emphasized the importance of immediate bystander CPR in saving lives after an out-of-hospital cardiac arrest and encourage year-round CPR training and awareness in all countries.^1^

Sudden cardiac arrest (SCA) is a life-threatening condition characterized by the abrupt cessation of cardiac activity, resulting in unresponsiveness, absence of normal breathing, and lack of circulation.^1^ Survival rates following SCA depend greatly on the actions taken by individuals nearby, such as immediately activating Emergency Medical Services (EMS), initiating cardiopulmonary resuscitation (CPR), and promptly using an automated external defibrillator (AED). These interventions can significantly improve the chances of survival for those experiencing SCA. The prompt administration of cardiopulmonary resuscitation (CPR) can greatly increase the likelihood of survival following cardiac arrest, potentially doubling or tripling the chances of survival.^2^ Enhancing CPR training can be achieved through various methods, such as classroom-based, online, and hands-on training. Incorporating CPR training into educational curricula and making CPR certification mandatory for higher education can ensure a wider reach of life-saving knowledge and skills.

Public access defibrillation (PAD) means availability of automated external defibrillators (AEDs) in public or private settings where large groups of people gather or where individuals at high risk for heart attacks reside.^3^ The widespread dissemination of public-access AEDs in Japan has resulted in a greater likelihood of laypersons administering early defibrillation, leading to an increase in the one-month survival rate with minimal neurologic impairment.^4^ In Stockholm, Sweden, where public access defibrillators (PADs) were available for out-of-hospital cardiac arrest (OHCA), a remarkable 70% of patients survived.^5^ Therefore, in addition to CPR training, the installation of AEDs in public places can significantly enhance the chances of survival for those experiencing sudden cardiac arrest (SCA).

However, increasing AED accessibility in India faces challenges such as high costs, maintenance requirements, and lack of public awareness regarding the crucial role that AEDs play in cases of sudden cardiac arrest (SCA). To address these challenges, the government should prioritize AED programs in villages and gradually expand to urban areas. Telephone cardiopulmonary resuscitation (TCPR) programs tailored to laypersons can also be introduced, particularly in developing nations like India, to empower individuals to provide life-saving assistance during SCA.

In conclusion, improving CPR training and increasing AED availability in public areas can effectively tackle sudden cardiac arrest cases in India. Collaboration between government agencies, private organizations, and the public is crucial for success. Further research is needed to evaluate the effectiveness of AED programs in India. By collectively addressing these challenges, we can enhance survival rates for SCA and create a healthier, more resilient society.

## Declaration of Competing Interest

The authors declare that they have no known competing financial interests or personal relationships that could have appeared to influence the work reported in this paper.
